# In Vitro Modeling of Paraxial Mesodermal Progenitors Derived from Induced Pluripotent Stem Cells

**DOI:** 10.1371/journal.pone.0047078

**Published:** 2012-10-24

**Authors:** Hidetoshi Sakurai, Yasuko Sakaguchi, Emi Shoji, Tokiko Nishino, Izumi Maki, Hiroshi Sakai, Kazunori Hanaoka, Akira Kakizuka, Atsuko Sehara-Fujisawa

**Affiliations:** 1 Department of Growth Regulation, Institute for Frontier Medical Sciences, Kyoto University, Kyoto, Japan; 2 Department of Clinical Application, Center for iPS Cell Research and Application, Kyoto University, Kyoto, Japan; 3 Laboratory of Functional Biology, Graduate School of Biostudies, Kyoto University, Kyoto, Japan; 4 Molecular Embryology, Department of Bioscience, School of Science, Kitasato University, Kanagawa, Japan; William Harvey Research Institute, Barts and The London School of Medicine and Dentistry, Queen Mary University of London, United Kingdom

## Abstract

Induced pluripotent stem (iPS) cells are generated from adult somatic cells by transduction of defined factors. Given their unlimited proliferation and differentiation potential, iPS cells represent promising sources for cell therapy and tools for research and drug discovery. However, systems for the directional differentiation of iPS cells toward paraxial mesodermal lineages have not been reported. In the present study, we established a protocol for the differentiation of mouse iPS cells into paraxial mesodermal lineages in serum-free culture. The protocol was dependent on Activin signaling in addition to BMP and Wnt signaling which were previously shown to be effective for mouse ES cell differentiation. Independently of the cell origin, the number of transgenes, or the type of vectors used to generate iPS cells, the use of serum-free monolayer culture stimulated with a combination of BMP4, Activin A, and LiCl enabled preferential promotion of mouse iPS cells to a PDGFR-α^+^/Flk-1^−^ population, which represents a paraxial mesodermal lineage. The mouse iPS cell-derived paraxial mesodermal cells exhibited differentiation potential into osteogenic, chondrogenic, and myogenic cells both *in vitro* and *in vivo* and contributed to muscle regeneration. Moreover, purification of the PDGFR-α^+^/KDR^−^ population after differentiation allowed enrichment of human iPS cell populations with paraxial mesodermal characteristics. The resultant PDGFR-α^+^/KDR^−^ population derived from human iPS cells specifically exhibited osteogenic, chondrogenic, and myogenic differentiation potential *in vitro*, implying generation of paraxial mesodermal progenitors similar to mouse iPS cell-derived progenitors. These findings highlight the potential of protocols based on the serum-free, stepwise induction and purification of paraxial mesodermal cell lineages for use in stem cell therapies to treat diseased bone, cartilage, and muscle.

## Introduction

Embryonic stem (ES) cells have been investigated both as an experimental tool for developmental biology and as a source of cell-based therapies due to their potential for self-renewal and differentiation into all cell lineages. Novel ES cell-like pluripotent stem cells, termed induced pluripotent stem (iPS) cells, have been generated from mouse [1] and human [2], [3] somatic cells by the introduction of 4 transcription factors. These iPS cells have opened the gateway for cell transplantation-based regenerative medicine by overcoming the ethical argument against human ES cells [4]. The original technology to generate iPS cells depended on the stable integration of 4 transgenes. However, the use of non-integrative vectors for gene transfer [5] or replacement of the *c-Myc* oncogene with other safer genes such as *L-Myc* [6] or *Glis-1* [7] permits generation of iPS cells that lack transgenes or oncogenes. Such technical advancements reduce the risk of tumorigenesis that results from reactivation of *c-Myc* transgenes. However, there is still a risk of teratoma formation, derived from residual undifferentiated cell populations after transplantation of differentiated iPS cells. Thus, efficient differentiation of iPS cells into the progenitor cells of interest and their maximal purification is required before transplantation. Moreover, suitable differentiation markers should be used to determine the signaling mechanisms that govern pluripotent stem cell differentiation toward specific lineages, so that recombinant proteins and small molecules can be used to direct differentiation.

Previously, we employed a murine ES cell *in vitro* differentiation culture system to show that expression of platelet-derived growth factor receptor α (PDGFR-α) allows efficient identification of paraxial mesodermal progenitors in combination with negative selection of Flk-1 expression–a lateral mesodermal marker [8]. The expression of PDGFR-α was detected in the paraxial mesoderm and somites as well as in neural tube and future spinal cord during mouse embryogenesis [9], [10]. Analysis of the *in vitro* fate of ES cell-derived PDGFR-α^+^/Flk-1^−^ cells demonstrated their potential to differentiate into osteocytes, chondrocytes, and skeletal muscle cells, which are derivatives of somites [8], [11]. We also showed that mouse ES cells can be directed toward the paraxial mesodermal lineage by a combination of bone morphogenetic protein (BMP) [12] and Wnt [13] signaling under chemically-defined conditions [14]. However, it is not known whether iPS cells also have the potential to give rise to paraxial mesodermal lineages by stimulating BMP and Wnt signaling cascades.

In the present study, we show that BMP4 and LiCl, which activate Wnt signaling, promote differentiation of both mouse iPS and ES cells to paraxial mesodermal lineages under serum-free conditions. However, unlike mouse ES cells, the self-renewal and differentiation of mouse iPS cells to paraxial mesodermal lineages is highly dependent on Activin A [15], which prevents apoptosis of mouse iPS cells in serum-free condition. In this serum-free differentiation system, mouse iPS cells efficiently differentiate into PDGFR-α^+^/Flk-1^−^ paraxial mesodermal progenitors and, to a lesser extent, into PDGFR-α^+^/Flk-1^+^ immature and PDGFR-α^−/^Flk-1^+^ lateral mesodermal progenitors. The iPS cell-derived paraxial mesodermal progenitors exhibit osteogenic, chondrogenic, and myogenic differentiation potential both *in vitro* and *in vivo*. Moreover, sorting of PDGFR-α-positive and KDR [16] (a human homolog of mouse Flk-1)-negative populations also allows enrichment of paraxial mesodermal progenitors in induced human iPS cells which give rise to osteogenic, chondrogenic and myogenic cells *in vitro*.

## Results

### Activin A is an Essential Factor for Paraxial Mesodermal Differentiation of Mouse iPS Cells

**Figure 1:**
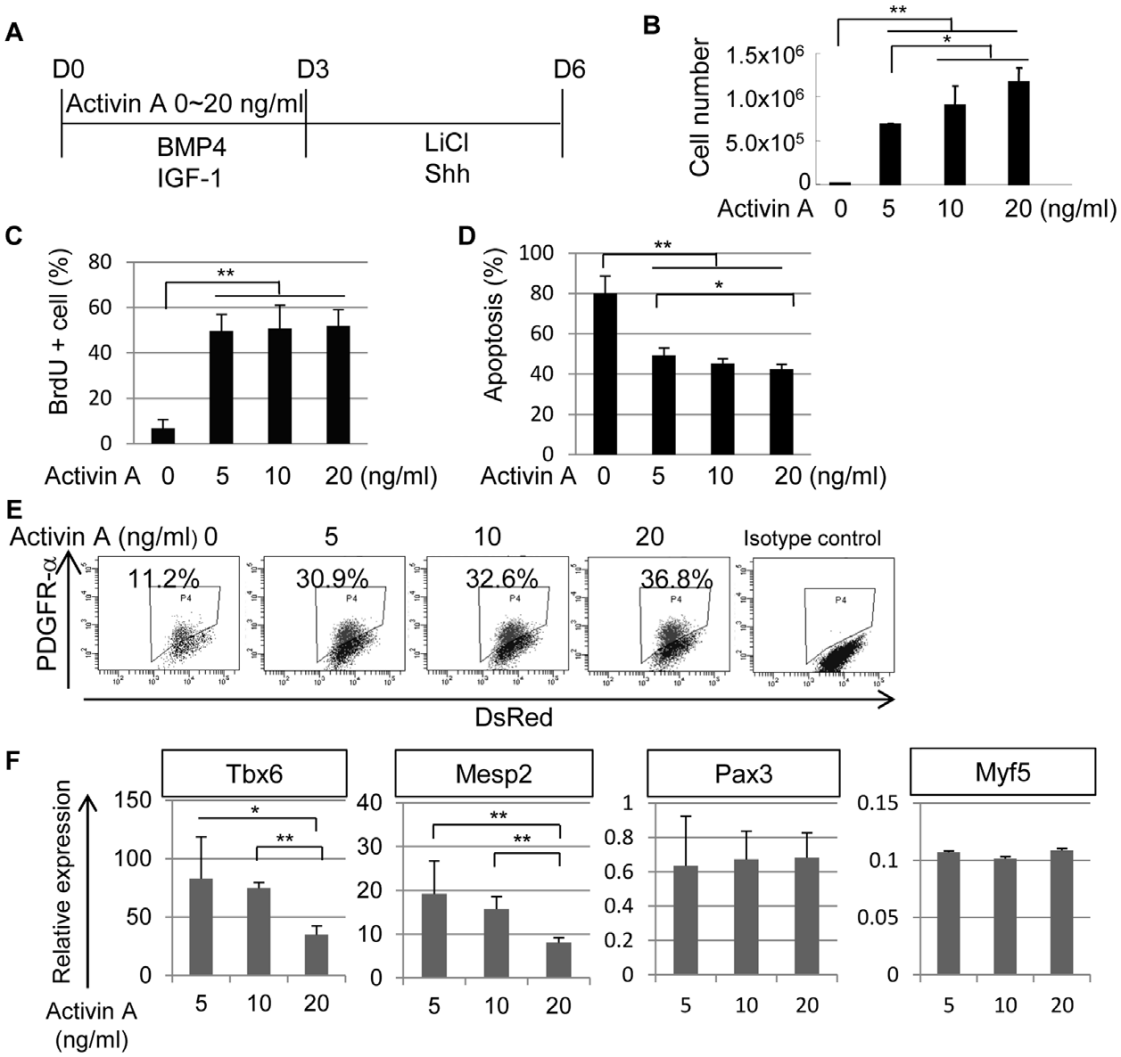
Effects of Activin A on paraxial mesodermal differentiation of mouse iPS cells. (**A**) A scheme for paraxial mesodermal differentiation of mouse iPS cells with different concentrations of Activin A from day 0 (D0) to day 3 (D3). Activin A was administrated from D0 to D3 at a concentration of 0–20 ng/ml. The cultures also contained BMP4 (10 ng/ml), IGF-1 (10 ng/ml), LiCl (5 mM), and Shh (10 ng/ml). The cells were analyzed on day 6 (D6) in (**B**), (**E**), and (**F**), or on day 1 (D1) in (**C**) and (**D**). (**B**) Total number of mouse iPS cells after differentiation with the protocol shown in (**A**) (n = 3). (**C**) Proliferation of differentiated mouse iPS cells on D1 assessed by BrdU assay (n = 3). (**D**) Apoptosis of differentiated mouse iPS cells on D1 assessed by a proportion of Propidium Iodide (PI) positive/AnnexinV positive cell (n = 3). (**E**) Dose-dependent induction of PDGFR-α by Activin A in mouse iPS cell differentiation culture. The percentage indicates the proportion of PDGFR-α^+^ cells (n = 3). (**F**) Gene expression profiles of PDGFR-α^+^ cells in Activin A-induced cultures (n = 3). The expression level of *Tbx6* and *Mesp2* genes was reduced in a dose-dependent manner. *p<0.05, **p<0.01 between selected two samples.

To establish chemically defined conditions for paraxial mesodermal differentiation *in vitro*, iPS cells must be cultured without feeder cells. Thus, the growth-factor requirement of mouse iPS cells during paraxial mesodermal differentiation was analyzed by performing an *in vitro* differentiation study with various doses of growth factors in chemically defined culture conditions. First, we assessed the effect of Activin A–a member of the transforming growth factor beta super family–during the first 3 days of differentiation (Fig. 1A). Differentiation of iPS cells without Activin A resulted in minimal proliferation/survival in the absence of feeder cells (Fig. 1B). However, the addition of Activin A dramatically enhanced cell number in a dose-dependent manner (Fig. 1B). Even low dose addition of Activin A supported efficient cell proliferation (Fig. 1C). Because we observed large amount of cell death in this serum-free condition, we assessed apoptosis at 24 hour after induction. Although, the serum-free condition caused apoptosis in more than 80% of iPS cells, addition of Activin A prevented apoptosis considerably (Fig. 1D). Previously, we have demonstrated that mouse ES cells can differentiate into paraxial mesoderm only with an addition of BMP4 in serum-free conditions [14]. Therefore, we compared the response of mouse iPS cells to Activin A with that of mouse ES cells. Surprisingly, mouse ES cells showed fewer apoptosis even in the absence of Acitivin A (Fig. S1A). Mouse ES cells showed higher expression level of endogenous *Nodal* [17] transcription than mouse iPS cells at 24 after induction (Fig. S1B), suggesting that mouse ES cells could survive in serum-free condition producing Nodal signaling. On the other hand, expressions of early mesoendodermal or mesodermal markers, such as *Mixl1* [18], *Gsc* [19], *T* [20] and *Eomes* [21], on day3 were enhanced by administration of Activin A in both mouse ES cells and mouse iPS cells (Fig. S1C), suggesting that Activin-Nodal signal directed differentiation toward mesodermal cells in both types of pluripotent stem cells.

Subsequently, paraxial mesodermal differentiation was assessed by the expression of PDGFR-α [8]. The addition of 5 ng/ml Activin A resulted in an increase in the number of cells expressing PDGFR-α to over 30%, and higher doses of Activin A increased the percentage of PDGFR-α^+^ cells in a dose-dependent manner (Fig. 1E).

The PDGFR-α^+^ cells were sorted and the gene expression level of mesodermal markers in each PDGFR-α^+^ population was analyzed by quantitative real-time RT-PCR. In Activin A-depleted conditions, there were not enough PDGFR-α^+^ cells to allow evaluation of mesodermal induction by RT-PCR analysis. While all samples were PDGFR-α^+^, the gene expression levels of paraxial mesodermal markers (*Tbx6* [22] and *Mesp2* [23]) varied with the dose of Activin A– expression was high in preparations treated with the lowest dose of Activin A and low in those treated with higher doses (Fig. 1F). In contrast, Activin A did not affect the expression level of late mesodermal markers such as the dermomyotome marker *Pax3* [24] and the myotome marker *Myf5* [25] (Fig. 1F). Thus, the addition of high doses of Activin A may alter the characteristics of the PDGFR-α^+^ population in mouse iPS cells. Since further lower dose of Activin A than 5 ng/ml did not influence the induction of paraxial mesodermal differentiation significantly (Fig. S1D), we used 5–20 ng/ml Activin A in further experiments because of dose-dependent proliferation/survival rates of iPS cells.

### BMP4 Enhances Cell Proliferation and Affects Lineage-specific Gene Expression during Mouse iPS Cell Differentiation

**Figure 2:**
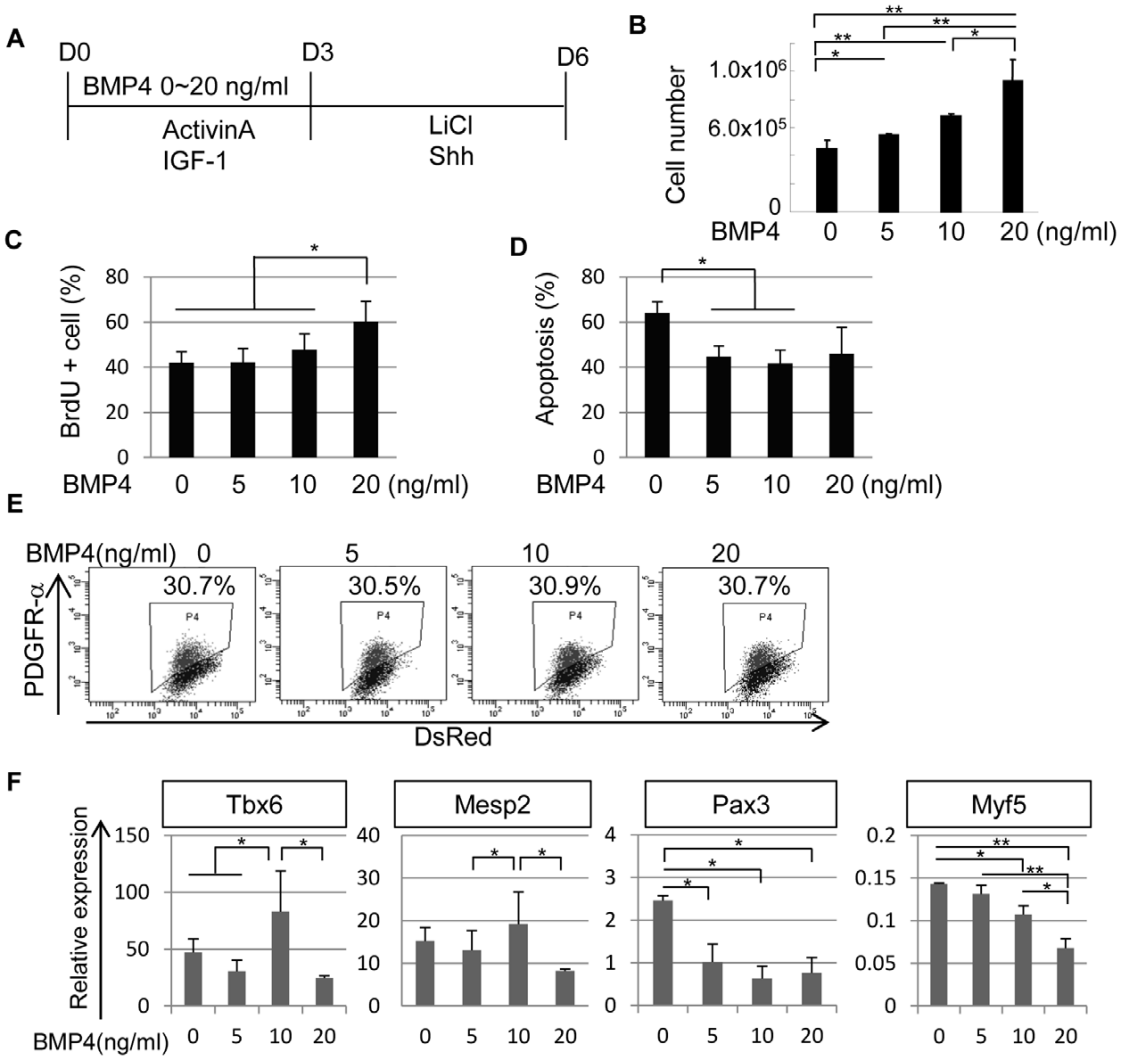
Effects of BMP4 on paraxial mesodermal differentiation of mouse iPS cells. (**A**) A scheme for the paraxial mesodermal differentiation of mouse iPS cells with different concentrations of BMP4 from D0 to D3. BMP4 was administrated from D0 to D3 at a concentration of 0–20 ng/ml. The cultures also contained Activin A (5 ng/ml), IGF-1 (10 ng/ml), LiCl (5 mM), and Shh (10 ng/ml). The cells were analyzed on D6 in (**B**), (**E**), and (**F**), or on day 3 (D3), as shown in (**C**) and (**D**). (**B**) Total number of mouse iPS cells after differentiation with the protocol shown in (**A**) (n = 3). (**C**) Proliferation of differentiated mouse iPS cells on D3 assessed by BrdU assay (n = 3). (**D**) Apoptosis of differentiated mouse iPS cells on D3 assessed by a proportion of PI positive/AnnexinV positive cell (n = 3). (**E**) The expression of PDGFR-α in mouse iPS cell differentiation culture. The percentage indicates the proportion of PDGFR-α^+^ cells (n = 3), which was not affected by BMP4. (**F**) Gene expression profiles of PDGFR-α^+^ cells in BMP4-induced cultures (n = 3). The expression of *Tbx6* and *Mesp2* was higher at a concentration of 10 ng/ml BMP4, whereas the expression of *Pax3* and *Myf5* was reduced at higher doses of BMP4. *p<0.05, **p<0.01 between selected two samples.

We subsequently assessed the effect of BMP4 during the first 3 days of differentiation under chemically defined culture conditions in the presence of Activin A (Fig. 2A). The addition of BMP4 enhanced cell number in a dose-dependent manner (Fig. 2B). While the addition of high dose of BMP4 enhanced cell proliferation (Fig.2C), the absence of BMP4 resulted in large apoptosis **(** Fig. 2D). These two functions of BMP4 administration cooperated to increase cell number in a dose-dependent manner.

However, the generation of the PDGFR-α^+^ population was unaffected by BMP4 treatment (Fig. 2E). The expression of paraxial mesodermal markers (*Tbx6* [22] and *Mesp2* [23]) was high when cells were induced with a medium dose (10 ng/ml) of BMP4 (Fig. 2F). In contrast, the addition of a high dose of BMP4 reduced the expression level of myogenic mesodermal markers, *Pax3* [24] and *Myf5* [25] (Fig. 2F). Down-regulation of *Myf5* by high-dose BMP4 administration is consistent with the evidence that BMP4 acts as an inhibitor for expression of myogenic regulatory genes *Myf5* and *MyoD* in mouse embryogenesis [26].

### Wnt Signaling Enhances Paraxial Mesodermal Differentiation of Mouse iPS Cells

**Figure 3:**
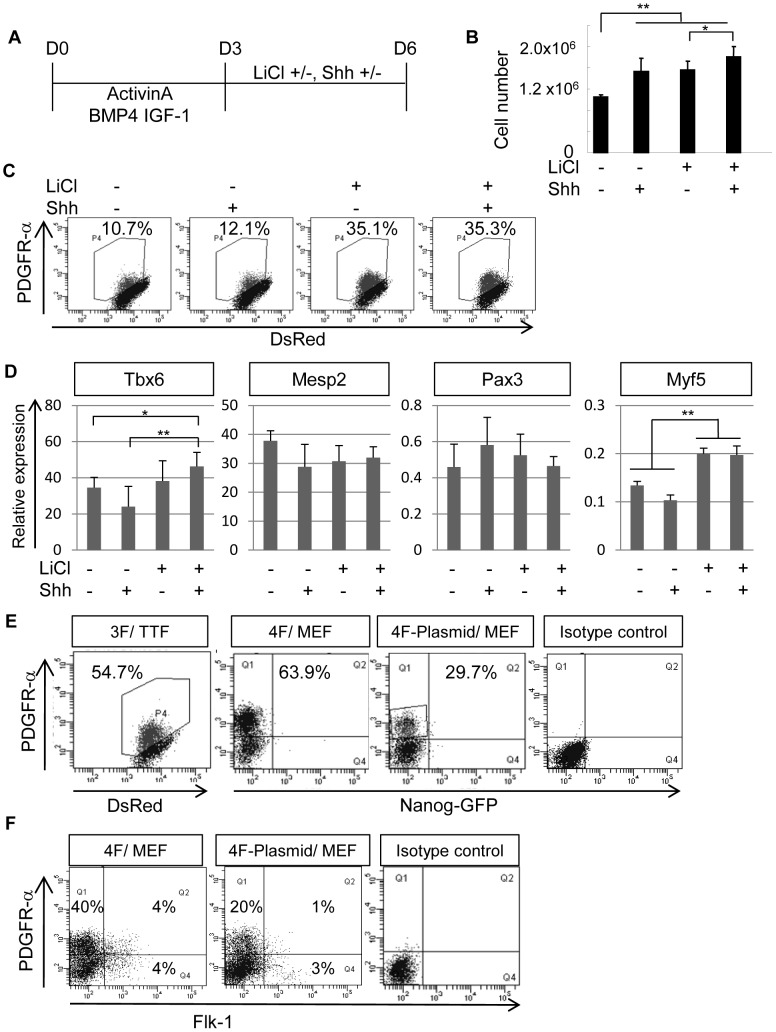
Effects of LiCl and Shh on paraxial mesodermal differentiation of mouse iPS cells. (**A**) A scheme for paraxial mesodermal differentiation of mouse iPS cells using various combinations of LiCl and Shh from D3 to D6. The cells were analyzed on D6 in (B–F). The cultures also contained Activin A (5 ng/ml), BMP4 (10 ng/ml), and IGF-1 (10 ng/ml). (**B**) Total number of mouse iPS cells after differentiation with the protocol shown in (**A**) (n = 3). Both LiCl and Shh enhanced proliferation of mouse iPS cells. (**C**) The expression of PDGFR-α in mouse iPS cells after differentiation. The percentage indicates the proportion of PDGFR-α^+^ cells (n = 3). LiCl prominently induced generation of PDGFR-α^+^ cells. (**D**) Gene expression profiles of PDGFR-α^+^ cultured as shown in (**A**) (n = 3). LiCl treatment enhanced expression of *Myf-5*. (**E**) Differentiation of various types of mouse iPS cell clones into PDGFR-α^+^ cells in serum-free induction culture. 3F/TTF: iPS cells induced by 3 factors (Oct3/4, Sox2, and Klf4) using retroviral transduction from tail-tip fibroblasts. 4F/MEF and 4F-Plasmid/MEF: iPS cells induced by 4 factors (Oct3/4, Sox2, Klf4, and c-Myc) from MEFs using retroviral transduction (4F/MEF), or plasmid transduction (4F-Plasmid/MEF), respectively. The percentage indicates the proportion of PDGFR-α^+^ cells (n = 3). (**F**) Expression profiles of 2 mesodermal markers, PDGFR-α and Flk-1, after mouse iPS cell differentiation. Up to 90% of PDGFR-α^+^ cells cultured under these conditions were PDGFR-α^+^/Flk-1^−^ paraxial progenitors. PDGFR-α^+^/Flk-1^+^ and PDGFR-α^−/^Flk-1^+^ populations were barely induced under these conditions. *p<0.05, **p<0.01 between selected two samples.

The effects of growth factors involved in somitogenesis during mouse development were assessed. During the last 3 days of differentiation in chemically defined culture conditions, mouse iPS cells were cultured in the presence or absence of LiCl and Sonic hedgehog (Shh), respectively (Fig. 3A). The suitable dose of LiCl and Shh were determined by induction level of PDGFR-α^+^ cells, respectively (Fig. S2A and S2B). LiCl enhances Wnt signaling by translocation of β-catenin from cytoplasm to nucleus (Fig. S2C) followed by inhibiting the activity of GSK3β [27]. The addition of both LiCl and Shh to the chemically defined medium enhanced cell number (Fig. 3B). The increase of cell number was mainly due to effects of Shh in cell proliferation (Fig. S2D), whereas the addition of LiCl inhibited apoptosis (Fig. S2E). While the addition of Shh did not influence generation of PDGFR-α^+^ cells in differentiation culture, LiCl prominently induced a PDGFR-α^+^ cell population (Fig. 3C). Moreover, the expression levels of *Tbx6* [22] were higher in the presence of LiCl, whereas the expression levels of *Mesp2* [23] and *Pax3* [24] remained unchanged under these conditions (Fig. 3D). The addition of LiCl, but not Shh, induced the expression of the myogenic mesodermal marker *Myf5* [25] (Fig. 3D). Thus, activation of Wnt signaling by LiCl promoted paraxial mesodermal differentiation of iPS cells, particularly to skeletal muscle cell lineages.

Given the aforementioned results, we optimized the culture conditions for paraxial mesodermal differentiation, as described in the Materials and Methods. The resulting differentiation protocol was applicable to all types of iPS cells examined, including those derived from adult tail-tip fibroblast (TTF) or mouse embryonic fibroblast (MEF) cells and those made by retroviral transgenesis with 3 or 4 factors or plasmid vectors (Fig. 3E). PDGFR-α^+^ cells are distinguishable by their piled-up morphology like somite in the differentiation culture (Fig. S3). Although PDGFR-α^+^ cells can be divided into 2 distinct populations by the co-expression of Flk-1, up to 90% of PDGFR-α^+^ cells cultured under these conditions consisted of PDGFR-α^+^/Flk-1^−^ paraxial progenitors (Fig. 3F). Thus, this result suggests that the PDGFR-α^+^ population on day 6 of differentiation includes mainly paraxial mesodermal progenitors rather than PDGFR-α^+^/Flk-1^+^ immature mesodermal progenitors [8].

### Mouse iPS Cell-derived Paraxial Mesodermal Progenitor Cells are able to Differentiate into Myocytes, Osteocytes, and Chondrocytes *in vitro*

**Figure 4:**
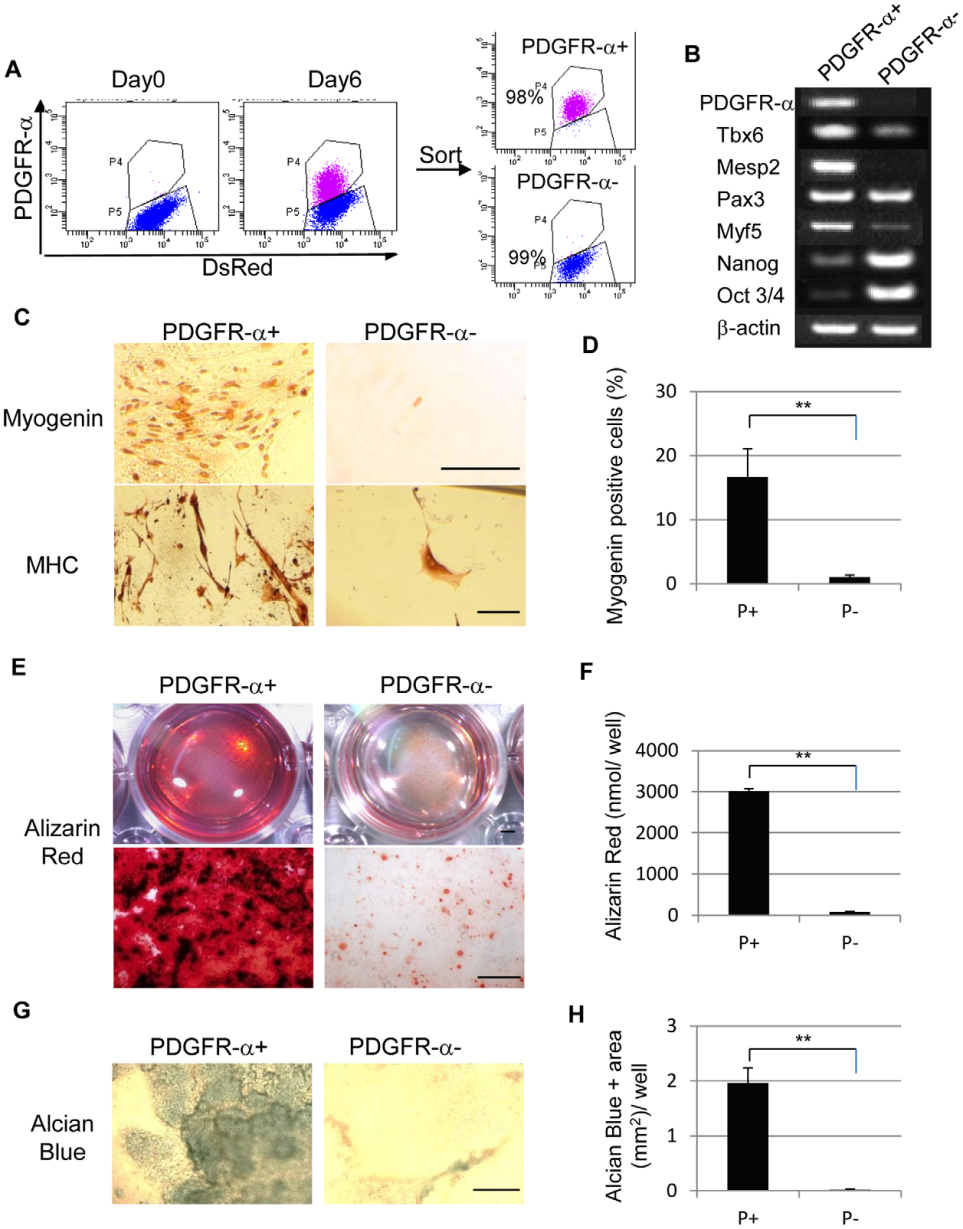
Differentiation potential of mouse iPS cell-derived paraxial mesodermal progenitors *in vitro*. (**A**) On day 6, differentiated iPS cells were sorted into PDGFR-α^+^ and PDGFR-α^−^ populations. (**B**) Gene expression profile for mesodermal and undifferentiated markers in PDGFR-α^+^ and PDGFR-α^−^ populations. (**C**) *In vitro* myogenic differentiation of sorted cells 7 days after differentiation. The differentiation of PDGFR-α^+^ cells, but not PDGFR-α^−^ cells, into mature myocytes is shown as myogenin^+^ cells with brown nuclear staining (upper panels) or as myosin heavy chain (MHC)-positive cells with brown cytosolic staining (lower panels). (**D**) The ratio of myogenin^+^ cells to the total number of cells that were Giemsa-positive in each well was counted. Approximately 16% of PDGFR-α^+^ cells were myogenin^+^ (n = 3). (**E**) *In vitro* osteogenesis of differentiated mouse iPS cells 28 days after osteocytic induction. The PDGFR-α^+^ population differentiated into osteocytes, producing an Alizarin Red-positive calcium matrix. The PDGFR-α^−^ population showed limited osteogenic potential, as indicated by faint calcium deposits (n = 3, each). (**F**) Quantification of Alizarin Red dyes in an osteogenic differentiation culture (n = 3). (**G**) *In vitro* chondrogenesis of differentiated mouse iPS cells 21 days after chondrocytic induction. The PDGFR-α^+^ population gave rise to Alcian Blue-positive chondrocytes. (n = 3, each) (**H**) Quantification of Alcian Blue positive area in a chondrogenic differemtiation culture (n = 3). The bars in (**C**) and (**G**) represent 100 µm, the bar in (**E upper**) represents 2 mm and the bar in (**E lower**) represents 200 µm. **p<0.01 between selected two samples.

We investigated the *in vitro* differentiation potential of mouse iPS cell-derived PDGFR-α^+^ cells to paraxial mesoderm descendants, such as myocytes, osteocytes, and chondrocytes. PDGFR-α^+^ cells isolated by FACS Aria displayed up to 98% purity (Fig. 4A). *Tbx6* positive, *Mesp2* positive paraxial mesodermal cells were mainly involved in the PDGFR-α^+^ population, while *Oct3/4* [28] positive, *Nanog* [29], [30] positive undifferentiated cells were mainly contained within the PDGFR-α^−^ population (Fig. 4B). In skeletal myogenic differentiation culture, myogenin-positive, myosin heavy chain (MHC)-positive mature myocytes were mainly detected in the PDGFR-α^+^ population, whereas very few myogenin-positive cells were derived from PDGFR-α^−^ cells (Fig. 4C). Moreover, approximately 16% of the PDGFR-α^+^ cells could give rise to myocytes, whereas only 1% of the PDGFR-α^−^ cells were myogenin positive (n = 3) (Fig. 4D). PDGFR-α^+^ cells differentiated into osteocytes containing an abundant calcium matrix, as revealed by Alizarin Red-positive staining in the well (Fig. 4E). On the other hand, the PDGFR-α^−^ population exhibited limited potential to form calcium-positive osteocytes, as indicated by reduced Alizarin Red staining (n = 3) (Fig. 4E). Quantification of total amount of Alizarin Red dyes in a well demonstrated that osteogenic cells were mainly contained in PDGFR-α^+^ population (Fig. 4F). Furthermore, during *in vitro* chondrogenesis, the PDGFR-α^+^ population formed an Alcian Blue-positive chondrocytic colony in the center of a high density micromass cell culture (Fig. 4G). In contrast, the PDGFR-α^−^ population did not form Alcian Blue-positive chondrocytes under the same conditions (n = 3) (Fig. 4G). Quantification of Alcian Blue-positive area in a well showed that chondrogenic cells were also contained in PDGFR-α^+^ population (Fig. 4H). These results indicate that PDGFR-α^+^ cells derived from mouse iPS cells had the potential to differentiate into paraxial mesodermal descendants *in vitro*, including skeletal myocytes, osteocytes, and chondrocytes. Thus, the mouse iPS cell-derived PDGFR-α^+^ cells displayed paraxial mesodermal characteristics.

### *In vivo* Differentiation Potential of Mouse iPS Cell-derived Paraxial Mesodermal Progenitor Cells

**Figure 5:**
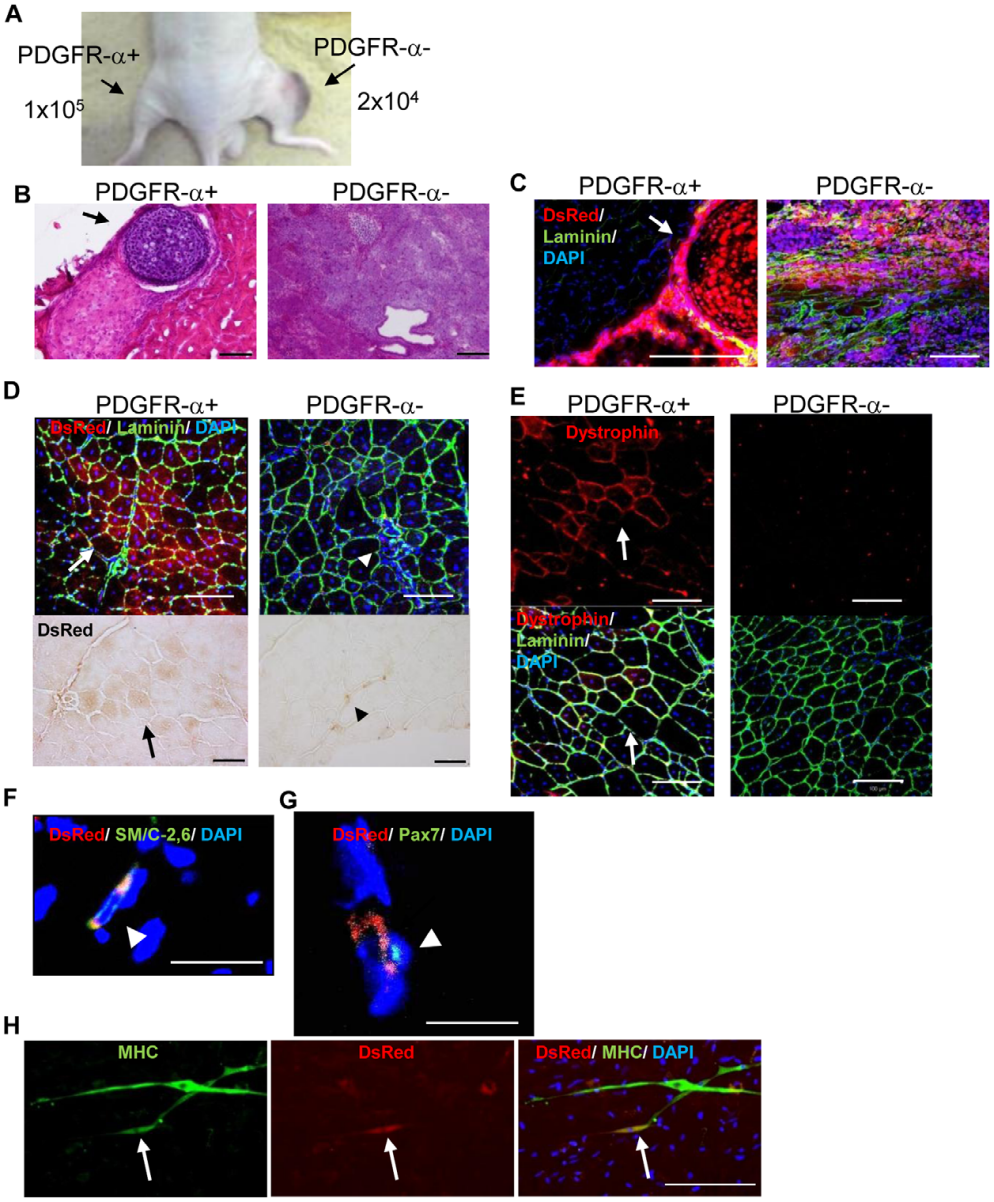
Differentiation potential of mouse iPS cell-derived paraxial mesodermal progenitors toward chondrocytes and myocytes *in vivo*. (**A**) Differentiated iPS cells were sorted into PDGFR-α^+^ and PDGFR-α^−^ populations on day 6. The resulting sorted cells were intramuscularly transplanted into the tibia anterior (TA) muscles of nude mice. Tumor formation was detected in muscle engrafted with the PDGFR-α^−^ population (n = 4). (**B**, **C**) The PDGFR-α^+^ population differentiated into chondrocytes *in vivo*. The iPS-DsRed cell-derived PDGFR-α^+^ or PDGFR-α^−^ populations within Matrigel were grafted into TA muscle. The PDGFR-α^−^ population formed a teratoma (**B**, right), and the PDGFR-α^+^ population formed ectopic cartilage (**B**, left; arrow). (**C**) The teratomas and ectopic cartilage were derived from engrafted cells that expressed DsRed. (**D**) Differentiation potential of the PDGFR-α^+^ population toward skeletal muscle *in vivo*. The iPS-DsRed cell-derived PDGFR-α^+^ or PDGFR-α^−^ populations were recultured on thermoreactive dishes for 24 h and harvested without enzymatic treatment. The harvested cells were directly transplanted into TA muscles of nude mice (n = 3, each). Immunohistochemical staining with anti-DsRed antibody was performed to detect engrafted cells. Upper panels: DsRed positive engrafted cells derived from PDGFR-α^+^ population fused with host myofibers (white arrow), while DsRed positive engrafted cells derived from PDGFR-α^−^ population located in interstitial area of host muscle (white arrowhead). Lower panels: DsRed expression was confirmed by HRP-based immunohistochemistry (black arrow and black arrowhead). (**E**–**G**) The PDGFR-α^+^ population differentiated to form dystrophin-positive muscle fibers in DMD-null mice. The harvested cells were directly transplanted into TA muscles of DMD-null mice (n = 3, each). (**E**) Immunohistochemical staining with anti-dystrophin antibody was performed to assess the contribution of engrafted cells to muscle regeneration. Dystrophin expression was detected at the injected site of PDGFR-α^+^ population engrafted muscle (white arrow), while no Dystrophin expression was observed in PDGFR-α^−^ population engrafted muscle. (**F**, **G**) To assess differentiation into satellite cells, immunohistochemical staining with SM/C-2.6 (**F**) and anti-Pax7 (**G**) antibodies was performed. DsRed-positive cells were able to differentiate into satellite cells (arrowheads). (**H**) DsRed-positive satellite cells differentiated into mature myocytes *in vitro* (arrow). The bars in (**B**), (**C**), (**D**), (**E**), and (**H**) represent 100 µm. The bars in (**F**) and (**G**) represent 10 µm.

To assess the potential of PDGFR-α^+^ cells to give rise to paraxial mesodermal descendants *in vivo*, we transplanted both PDGFR-α^+^ and PDGFR-α^−^ cells (derived from DsRed/iPS cells) into the tibial anterior (TA) muscle of immunodeficient mice. Four weeks after transplantation, tumor formation was only observed in TA muscle engrafted with PDGFR-α^−^ cells (n = 3) (Fig. 5A). The tumor was classified as a teratoma, which consisted of all 3 germ layers (Fig. 5B, right panel) and was derived from engrafted cells that expressed DsRed (Fig. 5C, right panel). Thus, these results suggest that PDGFR-α^−^ cells contain undifferentiated iPS cells. Engraftment of PDGFR-α^+^ cells resuspended in Matrigel caused ectopic cartilage formation in the TA muscle (n = 2 for 3 engraftments) (Fig. 5B, left panel). Ectopic cartilage was covered with a capsule and did not form part of the teratoma since it did not contain any other tissues. As shown in Fig. 5C (left panel), the ectopic cartilage was derived from engrafted cells that expressed DsRed. Transplantation into TA muscle of PDGFR-α^+^ cells resuspended in Matrigel resulted in the fusion of very few DsRed-positive cells with host myofiber (data not shown), as injection with Matrigel may have caused limited migration of engrafted cells. Therefore, we performed transplantation of fractionated cells in medium. However, no iPS cell-derived cells were detected following engraftment in medium, even in the case of the PDGFR-α^−^ population (data not shown), which may be due to the fact that FACS-sorted cells have low adhesion potential. In order to enhance the adhesion potential of the FACS-sorted cells, PDGFR-α^+^ and PDGFR-α^−^ cells were sorted and reseeded onto thermoresponsive culture dishes pre-coated with type IV collagen. Cells were harvested 24 h after re-culture by incubation at RT without enzymatic treatment. PDGFR-α^+^ and PDGFR-α^−^ cells were transplanted into the TA muscle of immunodeficient mice, and the engrafted tissues were analyzed 4 weeks after transplantation (n = 3). Detection of DsRed-positive myofibers indicated that the PDGFR-α^+^ cells had fused with host myofibers (Fig. 5D, white arrow). On the other hand, the PDGFR-α^−^ cells had never fused with myofibers and were observed in an interstitial area of muscles (Fig. 5D, white arrowhead). DsRed expression was confirmed by using an HRP-conjugated secondary antibody to exclude autofluorescence of mouse myofibers (Fig. 5D, black arrow and black arrowhead).

To assess the contribution of the engrafted PDGFR-α^+^ cells to myogenesis, we transplanted the cells into DMD-null mice, which is a model mouse for Duchenne muscular dystrophy (DMD) [31]. Four weeks after transplantation, DMD-null mice were sacrificed and dystrophin [32] expression in engrafted muscle was assessed by immunohistochemical analysis (n = 3). Dystrophin-positive myofibers were detected in TA muscle engrafted with PDGFR-α^+^ cells (Fig. 5E, white arrow), while no dystrophin expression was observed in the muscle engrafted with PDGFR-α^−^ cells (Fig. 5E, right panel), suggesting that the PDGFR-α^+^ cells have potential to differentiate into functional myogenic cells that fuse with host myofibers and produce dystrophin. The PDGFR-α^+^ cells also gave rise to muscle satellite cells, which are adult stem cells of striated muscle. The expression of SM/C-2.6 (a surface marker for quiescent satellite cells [33]) and of Pax7 [34] (a specific transcriptional factor for satellite cells) was detected in some DsRed-positive engrafted cells (Fig. 5F and 5G). Therefore, to assess whether these iPS cell-derived satellite cells were functional, we isolated single cells from engrafted TA muscle by enzymatic treatment and recultured them under the culture conditions of primary satellite cells. The iPS cell-derived DsRed-positive cells were able to differentiate into MHC-positive myofibers within host myofibers together with host myocyte (Fig. 5H, white arrow). These results suggest that PDGFR-α^+^ cells represent paraxial mesodermal progenitors that have the potential to differentiate into both cartilage and functional myogenic cells *in vivo*.

### The Combined Use of 2 Markers–PDGFR-α and KDR–allows Isolation of Paraxial Mesodermal Progenitors from Human iPS Cell Differentiation Culture

**Figure 6:**
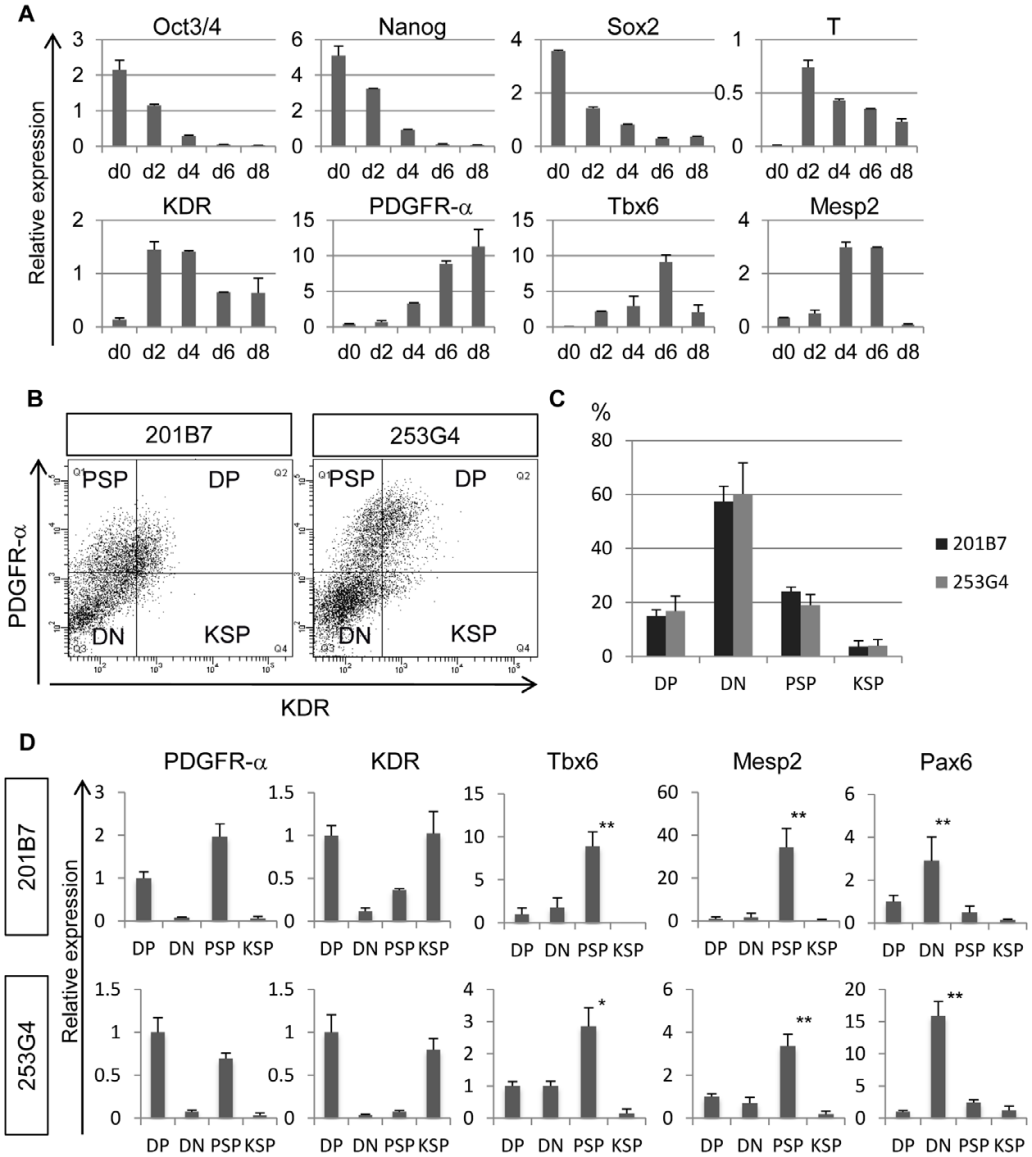
Modeling paraxial mesodermal differentiation of human iPS cells. (**A**) Time course of gene expression profile during differentiation of human iPS cells. (**B**) Expression profile of PDGFR-α and KDR in differentiated human iPS cells on day 6. DP, double-positive population; DN, double-negative population; PSP, PDGFR-α single-positive population; KSP, KDR single-positive population. (**C**) Proportion of each fractionated population of differentiated human iPS cells on day 6 (n = 3). About one third of cells were PDGFR-α positive, and around 20% of the total cells were classified in the PSP population. (**C**) Gene expression profiles of each population after differentiation of human iPS cells. *Tbx6* and *Mesp2* were dominantly expressed in the PSP population. *p<0.05, **p<0.01 PSP versus the other samples (Tbx6 and Mesp2). **p<0.01 DN versus the other samples (Pax6).

Next, we investigated paraxial mesodermal differentiation in human iPS cells. Although we attempted to use the same differentiation protocol as for mouse iPS cells, human iPS cells were not viable under these conditions. This may be due to the inability of human pluripotent stem cells to survive as single cells. Therefore, human iPS cells were dissociated into small clusters and differentiated in defined medium containing KSR. Gene expression profile of differentiated human iPS cells was analyzed during differentiation from day 0 to day 10 (Fig. 6A). The expression of undifferentiated markers, such as *Oct3/4* [28], *Nanog* [29], [30] and *Sox2* [35] gradually diminished along with differentiation (Fig. 6A). The expressions of early mesodermal marker *T* and lateral mesodermal marker *KDR* were detected after day 2 and gradually decreased as differentiation proceeded (Fig. 6A). The expression of *PDGFR-α* increased along with differentiation while paraxial mesodermal markers *Tbx6* and *Mesp2* were transiently activated at around day 4–6 (Fig.6A). Based on expression pattern of these genes, we tried to isolate paraxial mesodermal progenitors monitoring the expression of two mesodermal surface markers–PDGFR-α and KDR– at day6 differentiation. As a result, differentiated human iPS cells were separated into 4 fractions: PDGFR-α^+^/KDR^+^ (double positive; DP), PDGFR-α^+^/KDR^−^ (PDGFR-α single positive; PSP), PDGFR-α^−/^KDR^+^ (KDR single positive; KSP), and PDGFR-α^−/^KDR^−^ (double negative; DN) (Fig. 6B). As shown in Fig. 6C, the proportion of each fraction was similar in 2 distinct human iPS cell clones. Approximately one third of cells expressed PDGFR-α, and the PSP population rose to around 20% of cells in both iPS cell clones.

Previously, we reported that while the PSP population displayed paraxial mesodermal characteristics, the DP population displayed immature mesodermal characteristics during mouse ES cell differentiation [8]. Therefore, to assess the characteristics of each cell population, we performed quantitative PCR analysis of developmental markers (Fig. 6D). The expression levels of PDGFR-α and KDR were analyzed to confirm the purity of each FACs-sorted population. In both iPS cell clones, the paraxial mesodermal markers *Tbx6* and *Mesp2* were dominantly expressed in the PSP population. In contrast, the neuronal marker *Pax6* was specifically detected in the DN population, suggesting that neural lineage cells were negatively selected by the two mesodermal markers. Taken together, the gene expression profile indicates that the PSP population isolated after differentiation of human iPS cells displays paraxial mesodermal characteristics.

### The PDGFR-α^+^/KDR^−^ Population Differentiated from Human iPS Cells Exhibits Paraxial Mesodermal Characteristics with Differentiating Potentials into Skeletal Myocytes, Osteocytes, and Chondrocytes *in vitro*

**Figure 7:**
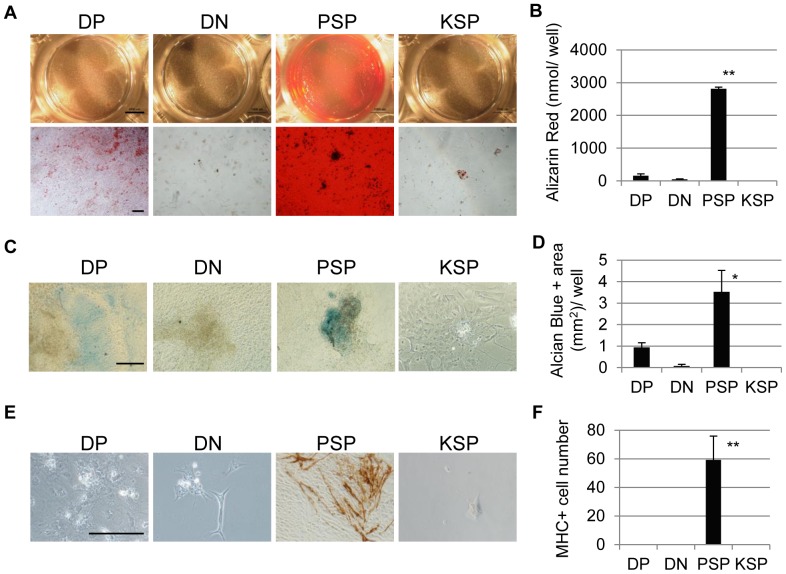
Differentiation potential of human iPS cell-derived mesodermal populations *in vitro*. (**A**) *In vitro* osteogenesis of differentiated human iPS cells 28 days after osteocytic induction. On day 6, differentiated human iPS cells were sorted into DP, DN, PSP and KSP populations. The PSP population differentiated into osteocytes, producing an Alizarin Red-positive calcium matrix. The DP population showed low osteogenic potential, as indicated by mild calcium deposits (n = 3, each). The DN and KSP populations had very low osteogenic potentials (n = 3, each). (**B**) Quantification of Alizarin Red dyes in an osteogenic differentiation culture (n = 3). (**C**) *In vitro* chondrogenesis of differentiated mouse iPS cells 21 days after chondrocytic induction. The PSP and DP populations gave rise to Alcian Blue-positive chondrocytes, while the DN and KSP populations had very low chondrogenic potentials. (n = 3, each) (**D**) Quantification of Alcian Blue positive area in a chondrogenic differemtiation culture (n = 3). (**E**) *In vitro* myogenic differentiation of sorted cells 14 days after differentiation. The differentiation of PPS cells, but not the other cells, into mature myocytes is shown as MHC^+^ cells with brown cytosolic staining (n = 3, each). (**F**) The number of MHC^+^ cells in a myogenic differentiation culture was counted (n = 3). The bar in (**A upper**) represents 4 mm, the bar in (**A lower**), (**C**) and (**E**) represents 100 µm. *p<0.05, **p<0.01 PSP versus the other samples.

Next, we investigated the *in vitro* differentiation potential of the PSP population derived from human iPS cells to paraxial mesoderm descendants, such as myocytes, osteocytes, and chondrocytes. Differentiated human iPS cells at Day6 were separated into 4 fractions–DP, DN, PSP and KSP– by FACS Aria and re-cultured for further differentiation. In osteogenic differentiation culture, Alizarin Red-positive calcium matrix was dominantly detected in the culture of PSP population, indicating that PSP cells have enough potential to differentiate into osteocytes (n = 3) (Fig. 7A). Quantification of total amount of Alizarin Red dyes in a well demonstrated that osteogenic cells were mainly contained within PSP population (Fig. 7B). In chondrogenic differentiation culture, both PSP population and DP population gave rise to Alcian Blue-positive chondrocytes (n = 3) (Fig. 7C). Quantification of Alcian Blue-positive area in a well showed that chondrogenic progenitors were mainly contained in PSP population (Fig. 7D). In myogenic differentiation culture, MHC-positive mature myocytes were selectively detected in the PSP population (n = 3) (Fig. 7E and 7F). These results indicate that PSP cells derived from human iPS cells have the potential to differentiate into three paraxial mesodermal descendants *in vitro*, including skeletal myocytes, osteocytes, and chondrocytes. Thus, these results suggest that the human iPS cell-derived PSP cells represent paraxial mesodermal progenitors.

## Discussion

Since Yamanaka and Takahashi first reported the generation of pluripotent ES cell-like cells–induced pluripotent stem (iPS) cells–from mouse fibroblasts by unique gene transfer-based nuclear reprogramming technology, many researchers have compared the similarities and differences between ES and iPS cells with respect to their pluripotency, undifferentiated states, genetic or epigenetic regulations, and differentiation potentials. For lateral mesodermal differentiation into cardiomyocytes, endothelial cells, and hematopoietic cells, mouse iPS cells have almost identical differentiation potential to mouse ES cells. In the present study, we demonstrated a potential of mouse ES and iPS cells for paraxial mesodermal differentiation, with the exception of the requirement of Activin A during iPS cell differentiation under serum-free conditions.

Previously, we demonstrated that BMP4 treatment was sufficient to promote commitment of mouse ES cells to the early primitive streak-type mesodermal lineage [14] and further their differentiation to paraxial mesodermal cell types. These findings are consistent with a previous finding, which showed that Wnt and Activin/Nodal are essential for the establishment of primitive streak-type mesodermal precursors during mouse ES cell differentiation and that BMP4 induces the endogenous activation of Nodal and Wnt pathways [36]. However, in the present study, we found that mouse iPS cells did not proliferate in the presence of BMP4 alone and that their survival and paraxial mesodermal differentiation required Activin A under serum-free conditions. The iPS cell clones used in this study display near complete reprogramming and show germline transmission in a chimeric assay [37], suggesting that the difference in the growth factor requirement between ES and iPS cells is not due to incomplete reprogramming of iPS cells. We rather consider that the requirement of Activin A by iPS cells is due to their intrinsic characteristics. More than 80% of the mouse iPS cells died by apoptosis in 24 hours after serum-free differentiation without Activin A, whereas only 10% of mouse ES showed apoptosis in the same culture condition. Mouse ES cells activate endogenous Nodal/Activin signaling in an autocrine fashion to proliferate under serum-free conditions [38]. Based on the finding that mouse iPS cells express less transcripts of the Nodal gene than mouse ES cells, we examined effects of Activin A on the serum-free culture of mouse iPS cells. As a result, we attained prominently enhanced survival and proliferation of mouse iPS cells in the presence of Activin A. On the other hand, the response to Activin A in early period of differentiation was quite identical between mouse ES and iPS cells in terms of increased expression of mesoendodermal genes [39]. Thus, mouse iPS cells are likely less capable of activating Nodal/Activin signaling, which is essential for their survival rather than for differentiation. Severe conditions, such as serum-free monolayer culture, should uncover differences between ES and iPS cells efficiently.

Although BMP4 was not essential for the induction of paraxial mesodermal progenitors from mouse iPS cells in the presence of both Activin A and LiCl and did not influence the proportion of PDGFR-α^+^ cells generated in cell culture, the addition of BMP4 affected the composition of PDGFR-α^+^ cells in a concentration-dependent manner. In particular, 10 ng/ml of BMP4 enhanced expression of paraxial mesodermal markers, whereas higher doses of BMP4 inhibited the expression of the myogenic marker *Myf5,* as consistent with previous reports [26]. In contrast, the addition of LiCl, which activates Wnt signaling via inhibition of GSK3β, dramatically increased the proportion of PDGFR-α^+^ cells in serum-free culture. Mesodermal gene expression patterns in the presence or absence of LiCl were almost identical, with the exception of elevated *Myf5* expression in the presence of LiCl. Therefore, these results suggest that Wnt signaling promotes paraxial mesodermal differentiation, particularly toward myogenic differentiation, during mouse iPS cell differentiation.

Although some differences in growth factor requirement between mouse ES cell and iPS cells were observed, the PDGFR-α^+^ population derived from mouse iPS cells was almost identical to that of ES cells with respect to their differentiation potential toward paraxial mesoderm descendants, such as osteocytes, chondrocytes, and myocytes. Particularly for myogenic differentiation, the iPS cell-derived PDGFR-α^+^ population contains myogenic progenitor cells that can give rise to satellite cells and contribute to regeneration of impaired muscle in DMD-null mice by the production of dystrophin. However, since the same PDGFR-α^+^ population induces ectopic cartilage formation, even following engraftment into muscle with Matrigel, it will be necessary to further dissect this population into committed myogenic and chondrogenic populations by using other markers.

In human iPS cell differentiation, PDGFR-α has been recognized as a segregation marker for isolating KDR^+^/PDGFR-α^−^ hematopoietic progenitors and KDR^+^/PDGFR-α^+^ cardiac progenitors among KDR^+^ mesodermal progenitors [40], [41]. In the present study, we demonstrated that PDGFR-α can be effectively used in combination with KDR negative selection to isolate paraxial mesodermal progenitor cells from human iPS cells after differentiation as well as in a mouse ES/iPS cell differentiation system. The human iPS cell-derived PDGFR-α^+^/KDR^−^ cells named PDGFR-α single positive (PSP) population significantly exhibited higher differentiation potentials to paraxial mesodermal descendants such as osteocytes, chondrocytes and myocytes than the other three populations. Whereas the protocols for osteogenic and chondrogenic differentiation of human iPS cells were similar to the protocols of mouse iPS cell, myogenic differentiation had to be customized for human iPS cells. The human iPS cell-derived PSP population did not differentiate to myocytes in the presence of serum or KSR, but did differentiate to myocytes in serum-free condition with additional supplement of LiCl and a TGF-β signal inhibitor SB-431542 [42]. However, many other cell types were still observed in this myogenic differentiation culture and efficiency of myogenesis still remained low. It will be necessary to explore further purification of committed myogenic lineage from PSP population by using other markers.

Finally, the isolation of paraxial mesodermal progenitors during human iPS cell differentiation will be a first step to purify osteogenic, chondrogenic and myogenic progenitors by their segregation from residual undifferentiated stem cells for use in stem cell therapies to treat diseased bone, cartilage, and muscle.

## Materials and Methods

### *In vitro* Cell Culture and iPS Cell Differentiation

Mouse iPS-MEF-Ng-20D-17 (iPS-Ng), iPS-MEF-Ng-492B-4 (iPS-plasmid), and iPS-TTF-DsRed-256H-18 (iPS-DsRed) iPS cells were kindly provided by Dr. S. Yamanaka and maintained as described previously [5], [43], [44]. Mouse fibroblasts were obtained from mouse tail-tip or embryo in strict accordance with the recommendations in the Regulation on Animal Experimentation at Kyoto University, and the protocol to produce the fibroblasts was approved by the Animal Research Committee of Kyoto University. For paraxial mesoderm differentiation, 1.6–2.4×10^5^ mouse iPS cells were plated onto a 6-cm dish coated with type IV collagen (Nitta Gelatin, Inc.) and differentiated in serum-free culture medium (SF-O3; Sanko Junyaku) supplemented with 0.2% bovine serum albumin (BSA), 0.1 mM 2-mercaptoethanol (2-ME), 5 ng/ml recombinant human Activin A (Peprotech), 10 ng/ml recombinant human IGF-1 (Peprotech), and 10 ng/ml recombinant human BMP4 (R&D Systems Inc., Minneapolis). After 3 days of culture, the medium was replaced with SF-O3 supplemented with 0.2% BSA, 0.1 mM 2-ME, 5 mM LiCl (Nacalai Tesque), and 10 ng/ml recombinant mouse Shh (R&D). Following 6 days of culture, paraxial mesodermal progenitor cells were obtained as PDGFR-α^+^ cells. For osteocytic and chondrocytic induction, paraxial mesodermal progenitor cells were sorted as described below and recultured as previously described [8]. Similarly, for myogenic differentiation, paraxial mesodermal progenitor cells were sorted as described below and recultured as described previously [14].

Human iPS cells (201B7 and 253G4) were also kindly provided by Dr. S. Yamanaka and maintained as previously described [2]. Human dermal fibroblasts used for generating 201B7 and 253G4 were purchased from Cell Applications, Inc. All human iPS cells were established according to procedures approved by Ethics Committee on Human Stem Cell Research of the Institute for Frontier Medical Sciences, Kyoto University. For mesodermal differentiation, feeder cells were depleted by treatment with 0.25% trypsin (Invitrogen), 0.1 mg/ml collagenase IV (Invitrogen), 1 mM CaCl_2_ (Nacalai Tesque), and 20% Knockout serum replacement (KSR; Invitrogen) at room temperature (RT) for 2 min. After feeder cell depletion, human iPS cells were washed twice with PBS(-), and an appropriate volume of differentiation medium, [alpha-minimum essential medium (αMEM; Nacalai Tesque) supplemented with 5% KSR and 0.1 mM 2-ME] was added to the culture dish. Human iPS cells were detached by scraping and dissociated into small clusters by pipetting, which were plated onto dishes coated with type I collagen (passage ratio, 1∶2). Human iPS cells were cultured in differentiation medium at 37°C with 5% CO_2_, and the differentiation medium was changed every 2 days. For osteocytic and chondrocytic induction, fractionated cells were sorted as described below and recultured as previously described [8]. For myogenic induction, the fractionated cells were sorted as described below and were re-cultured on collagen type I coated 24-well trays (AGC TECHNO GLASS) in SF-O3 with 5 mM LiCl, 10 ng/mL IGF-1, 10 ng/mL hepatocyte growth factor (HGF) (R&D systems) and 10 ng/mL basic fibroblast growth factor (bFGF) (Wako). Three days after re-culture, the medium was changed again to SF-O3 with 5 mM LiCl, 10 ng/mL IGF-1 and 5 µM SB-431542 (Stemgent Inc.). Four days later, the medium was changed again to SF-O3 with 10 ng/mL IGF-1, 5 µM SB-431542 and 10 ng/mL HGF and the cells were cultured for seven days.

### Antibodies, Cell Staining, FACS Analyses, and Cell Sorting

The rat monoclonal antibody (MAb) APA5 (anti-PDGFR-α) [9] was kindly provided by Dr. S. Nishikawa. Phycoerythrin-conjugated streptavidin (BD Pharmingen, San Diego, CA) was used to detect biotinylated-APA5 antibody. Allophycocyanin (APC)-conjugated AVAS12 (Anti-Flk-1) MAb was purchased from Biolegend.

Cultured cells were harvested and collected in 0.05% trypsin-EDTA (GIBCO, Grand Island, NY). Single-cell suspensions were stained as previously described [45] and analyzed or sorted with a FACS Aria (Becton, Dickinson and Company, Franklin Lakes, NJ).

### BrdU Assay

Differentiated iPS cells were labeled with 10 mM BrdU for four hours at day3 or day6. The BrdU Flow kit (BD Pharmingen) was used for detection of BrdU positive cells according to the manufacturer’s protocol.

### Apoptosis Assay

Differentiated iPS cells were harvested at day1, day3 or day6. The Annexin V : FITC Apoptosis Detection Kit I (BD Pharmingen) was used for detection of apoptotic cells according to the manufacturer’s protocol.

### Transplantation of iPS Cell-derived Mesodermal Progenitors into Mice

All mouse experiments were carried out according to protocols approved by the Animal Research Committee of Kyoto University. The PDGFR-α^+^ and PDGFR-α^−^ fractions were purified and collected by FACS Aria. Cells were resuspended in Matrigel (Invitrogen) or in αMEM supplemented with 10% fetal bovine serum (FBS; Invitrogen) and 100 µM 2-ME. Alternatively, collected cells were reseeded onto thermo-responsive culture dishes pre-coated with type IV collagen and were cultured in αMEM supplemented with 7% FBS, 10 ng/ml recombinant human IGF-1 and 100 µM 2-ME. Cells were harvested 24 h after reseeding by incubation at RT without enzymatic treatment and were resuspended in the culture media. For intra-muscular transplantation of immunodeficient mice, the tibia anterior (TA) muscle of a KSN nude mouse (Japan SLC, Inc., Hamamatsu, Japan) was treated with Cardiotoxin (CalBiochem) following diethyl ether anesthesia 24 h before transplantation, which consisted of the direct injection of 50 µl of cell suspension into the TA muscle of each mouse. For intra-muscular transplantation of a Duchenne muscular dystrophy (DMD)-model mouse, a DMD-null mouse was totally irradiated with 6 Gy of γ-rays and the TA muscle was treated with Cardiotoxin (CalBiochem) following diethyl ether anesthesia 24 hour before transplantation. For cell transplantation, 50 µl of cell suspension was directly injected into the TA muscle of each mouse. All mice used in this study were humanely sacrificed 28 days after transplantation and tissue samples were collected.

### Quantitative RT-PCR Analysis

Total RNA was isolated using Sepazol reagent (Nacalai Tesque) according to the manufacturer’s protocol. Residual genomic DNA was digested and removed with DNase I (Invitrogen). First-strand cDNA was synthesized using the Superscript III First-Strand Synthesis System (Invitrogen) for RT-PCR and oligo (dT) 12–18 primers. The cDNA was diluted with DNase-free water at a concentration of 10 ng/µl. Quantitative RT-PCR was performed using the PowerSYBR PCR kit (Applied Biosystems) according to the manufacturer’s instructions and a StepOne thermal cycler (Applied Biosystems). We used the *Rpl13a* and *β-actin* genes as invariant controls for mouse and human samples, respectively. For standardization of mouse iPS cell experiments, cDNA from E13.5 mouse embryo was defined as the control value (1.0). For human iPS cell experiments, we defined the value of the cDNA from a teratoma derived from 201B7 (Fig. 6A), or double positive (DP) (Fig. 6D) as the control value (1.0) for standardization. The primers are listed in Table S1.

### Histochemical Staining for Osteogenesis and Chondrogenesis

Alizarin Red and Alcian Blue staining was performed as previously described [46]. Briefly, cultured cells were fixed with 4% paraformaldehyde in PBS for 5 min at RT and subsequently washed twice with PBS for 5 min at RT. For calcium detection, fixed cells were incubated in 1% Alizarin Red S solution (Sigma) for 5 min at RT and subsequently washed 5 times with PBS at RT. For the detection of mucopolysaccharide in cartilage, fixed cells were incubated in 3% acetate solution for 5 min at RT and subsequently incubated in 1% Alcian Blue 8GX (Sigma) and 3% acetate solution for 30 min at RT. Finally, the cells were washed 5 times with 3% acetate solution and twice with PBS.

### Immunohistochemistry

Immunohistochemical analyses were performed as previously described [47], and the following antibodies were used: rabbit anti-DsRed (Clontech; 1∶5000), mouse anti-myogenin (Santa Cruz Biotechnology, Inc., Santa Cruz, CA; 1∶400), rabbit anti-dystrophin (Abcam; 1∶200), mouse anti-Pax7 (R&D; 1∶100), rat anti-laminin a2 (Alexis; 1∶150), rabbit anti-β-catenin (Sigma; 1∶500), goat anti-PDGFR-α (R&D; 1∶200) and rabbit anti-myosin heavy chain (R&D; 1∶400). A rat MAb satellite cell marker, SM/C-2.6 [33], was kindly provided Dr H. Fukada and used at a dilution of 1∶400. Horse radish peroxidase (HRP)-conjugated anti-mouse IgG (Chemicon), Alexa 488-conjugated anti-rat IgG (Invitrogen), Alexa 488-conjugated anti-mouse IgG (Invitrogen), Alexa 568-conjugated anti-rabbit IgG (Invitrogen), and Alexa 568-conjugated anti-goat IgG (Invitrogen) were used as secondary antibodies. For detection of Pax7, an anti-Pax7 antibody was directly labeled with Alexa-488 by using the Zenon system (Invitrogen).

### Animal Welfare

This study was carried out in strict accordance with the recommendations in the Regulation on Animal Experimentation at Kyoto University. The protocols in this study were approved by the Animal Research Committee of Kyoto University (Protocol number CiRA1-3 to Hidetoshi Sakurai). All injections were performed under anesthesia, and all efforts were made to minimize suffering. Mice were humanely sacrificed prior to tissue collection.

### Statistical Analysis

Differences between samples were assessed by using the Student’s two-tailed t test for independent samples.

## Supporting Information

Figure S1 **Responses to Activin A of mouse ES/iPS cells during mesodermal differentiation.** (**A**) Apoptosis of differentiated mouse ES cells on D1 assessed by a proportion of Propidium Iodide (PI) positive/AnnexinV positive cell (n = 3). (**B**) Nodal expression of differentiated ES and iPS cells on D1. More Nodal expression was observed in mouse ES cells than mouse iPS cells. (**C**) Expression level of early mesendodermal markers in mouse ES and iPS cells on D3 with (gray bars) or without (black bars) addition of Activin A (n = 3). Activin A treatment significantly activated expression of mesoendodermal genes in both mouse ES and iPS cells. (**D**) Gene expression profiles of PDGFR-α^+^ cells in lower dose treatment of Activin A (n = 3). The expression levels of *Tbx6* and *Mesp2* genes were not affected within those concentration of Activin A. *p<0.05, **p<0.01 between selected two samples.
(PDF)

Figure S2 **Responses to LiCl and Shh of mouse iPS cells during mesodermal differentiation.** (**A, B**) The expression of PDGFR-α in mouse iPS cells after differentiation. The gray bars indicates the percentage of PDGFR-α^+^ cells in various concentration of LiCl (**A**) or Shh (**B**) (n = 3, each). We choose suitable dose of LiCl or Shh by the proportion of PDGFR-α^+^ cells, as 5 mM LiCl or 10 ng/ml Shh, respectively. (**C**) LiCl acts as an inducer of Wnt signaling. The localization of β-catenin shifted from cytoplasmic region (upper panels) into nucleus (lower panels) after LiCl treatment. (**D**) Proliferation of differentiated mouse iPS cells on D6 assessed by BrdU assay (n = 3). Addition of Shh significantly promoted cell proliferation. (**E**) Apoptosis of differentiated mouse ES cells on D6 assessed by a proportion of PI positive/AnnexinV positive cell (n = 3). Addition of LiCl significantly reduced apoptosis of differentiated iPS cells. *p<0.05, **p<0.01 between selected two samples. The bar represents 50 µm.
(PDF)

Figure S3 **Morphology of PDGFR-α positive cells after mesodermal differentiation of mouse iPS cells.** PDGFR-α^+^ cells (red) are distinguishable by their prominent cellular clumping on D6 differentiation. The bars represent 100 µm.
(PDF)

Table S1 **Primes used for RT-PCR.** All forward primers are indicated in upper rows, and all reverse primers are indicated in lower rows.
(DOC)
