# Supplementary figures and images for: In Vitro Modeling of Paraxial Mesodermal Progenitors Derived from Induced Pluripotent Stem Cells
Source: PLoS One. 2012 Oct 24;7(10):e47078. doi: 10.1371/journal.pone.0047078 (PMC3480377; doi:10.1371/journal.pone.0047078)

Figure S1

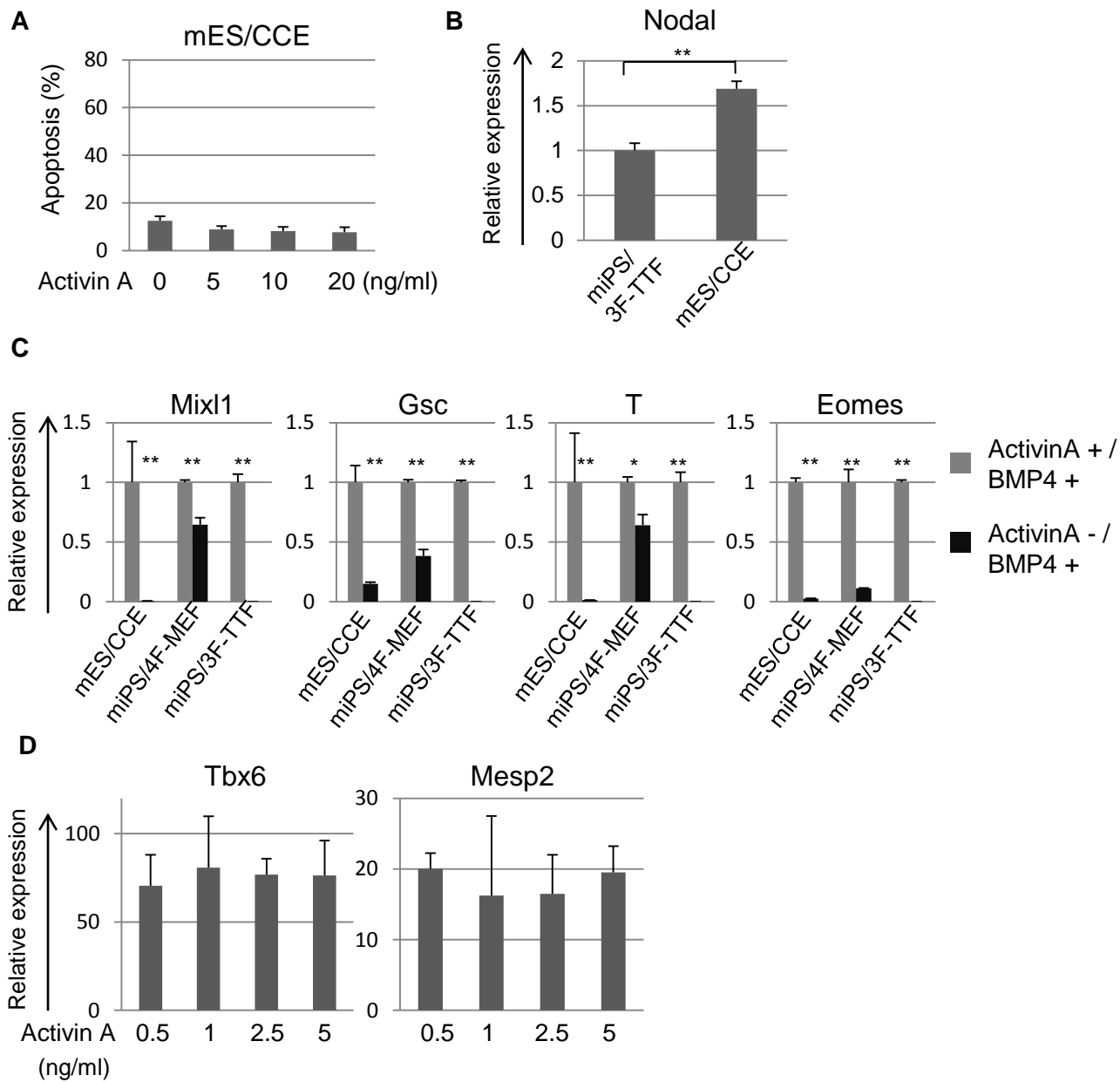

Supplement: Figure S1 — Responses to Activin A of mouse ES/iPS cells during mesodermal differentiation. (A) Apoptosis of differentiated mouse ES cells on D1 assessed by a proportion of Propidium Iodide (PI) positive/AnnexinV positive cell (n = 3). (B) Nodal expression of differentiated ES and iPS cells on D1. More Nodal expression was observed in mouse ES cells than mouse iPS cells. (C) Expression level of early mesendodermal markers in mouse ES and iPS cells on D3 with (gray bars) or without (black bars) addition of Activin A (n = 3). Activin A treatment significantly activated expression of mesoendodermal genes in both mouse ES and iPS cells. (D) Gene expression profiles of PDGFR-α+ cells in lower dose treatment of Activin A (n = 3). The expression levels of Tbx6 and Mesp2 genes were not affected within those concentration of Activin A. *p<0.05, **p<0.01 between selected two samples. (PDF) [file pone.0047078.s001.pdf]

Figure S2

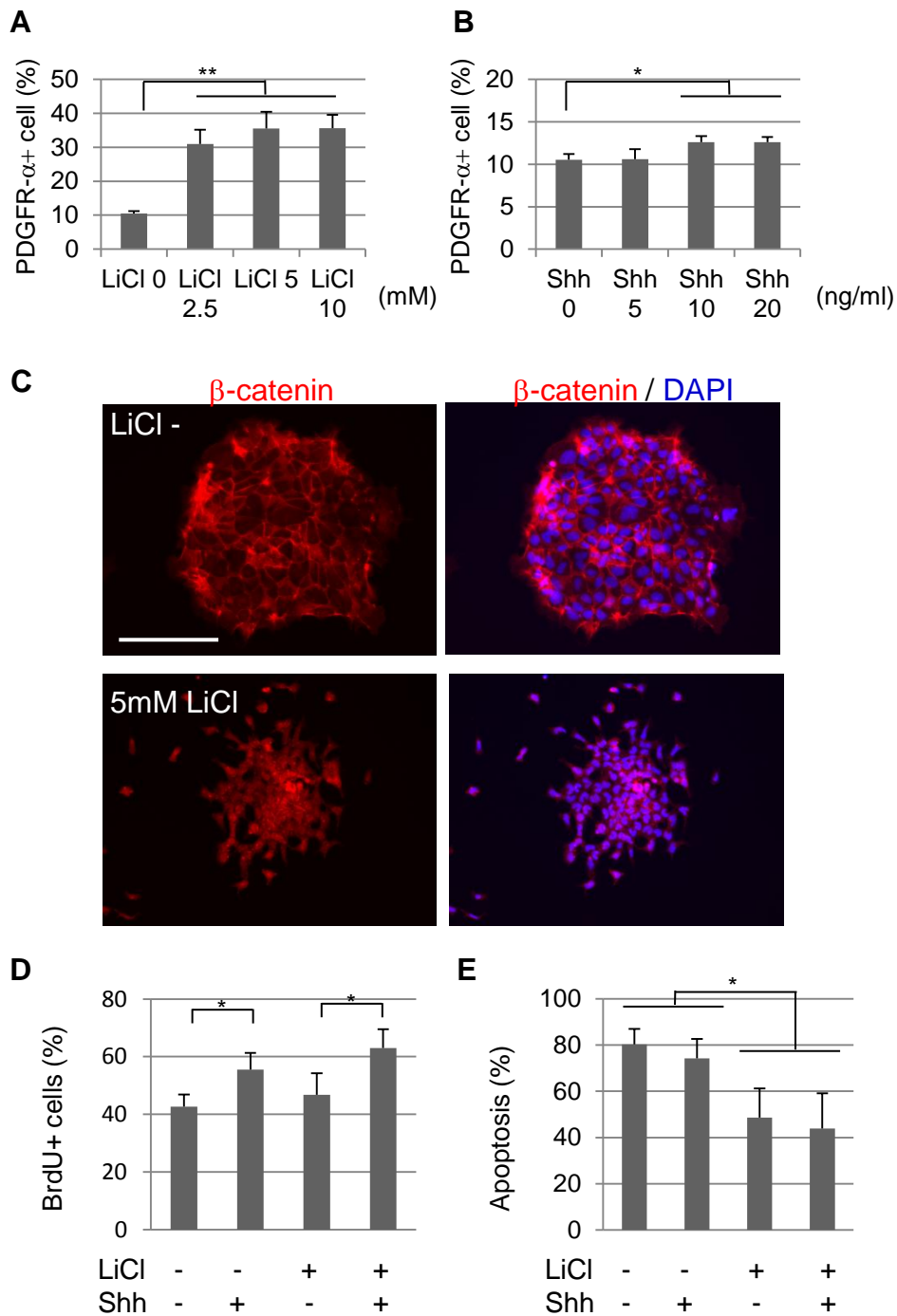

Supplement: Figure S2 — Responses to LiCl and Shh of mouse iPS cells during mesodermal differentiation. (A, B) The expression of PDGFR-α in mouse iPS cells after differentiation. The gray bars indicates the percentage of PDGFR-α+ cells in various concentration of LiCl (A) or Shh (B) (n = 3, each). We choose suitable dose of LiCl or Shh by the proportion of PDGFR-α+ cells, as 5 mM LiCl or 10 ng/ml Shh, respectively. (C) LiCl acts as an inducer of Wnt signaling. The localization of β-catenin shifted from cytoplasmic region (upper panels) into nucleus (lower panels) after LiCl treatment. (D) Proliferation of differentiated mouse iPS cells on D6 assessed by BrdU assay (n = 3). Addition of Shh significantly promoted cell proliferation. (E) Apoptosis of differentiated mouse ES cells on D6 assessed by a proportion of PI positive/AnnexinV positive cell (n = 3). Addition of LiCl significantly reduced apoptosis of differentiated iPS cells. *p<0.05, **p<0.01 between selected two samples. The bar represents 50 µm. (PDF) [file pone.0047078.s002.pdf]

Figure S3

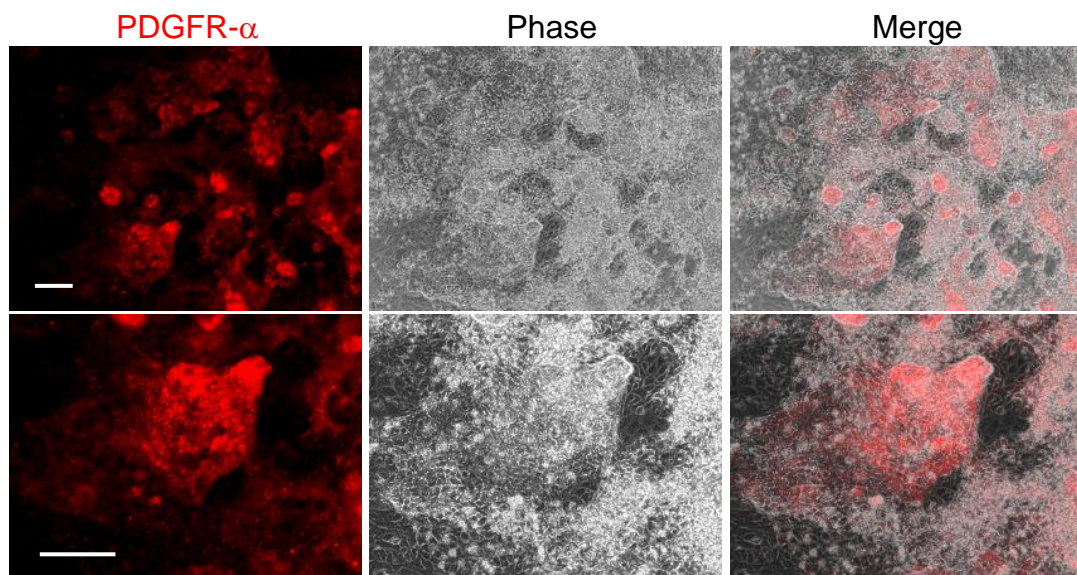

Supplement: Figure S3 — Morphology of PDGFR-α positive cells after mesodermal differentiation of mouse iPS cells. PDGFR-α+ cells (red) are distinguishable by their prominent cellular clumping on D6 differentiation. The bars represent 100 µm. (PDF) [file pone.0047078.s003.pdf]
