# Supplementary material for: In Vitro Modeling of Paraxial Mesodermal Progenitors Derived from Induced Pluripotent Stem Cells
Source: PLoS One. 2012 Oct 24;7(10):e47078. doi: 10.1371/journal.pone.0047078 (PMC3480377; doi:10.1371/journal.pone.0047078)
Supplement: Table S1 — Primes used for RT-PCR. All forward primers are indicated in upper rows, and all reverse primers are indicated in lower rows. (DOC) [file pone.0047078.s004.doc]

**Table S1** Primes used for RT-PCR

| Genes | Sequences |
| --- | --- |
| Human β-Actin | 5’- CACCATTGGCAATGAGCGGTTC -3’ |
|  | 5’- AGGTCTTTGCGGATGTCCACGT -3’ |
| Human PDGFR-α | 5’- GGCCCCATTTACATCATCAC -3’ |
|  | 5’- CATAGCTCCGTGTGCTTTCA -3’ |
| Human KDR | 5’- GCGATGGCCTCTTCTGTAAG -3’ |
|  | 5’- ACACGACTCCATGTTGGTCA -3’ |
| Human Pax6 | 5’- TGTCCAACGGATGTGTGAGT -3’ |
|  | 5’- TTTCCCAAGCAAAGATGGAC-3’ |
| Human Mesp2 | 5’- ACTGCCCCAAGATACAGTCG -3’ |
|  | 5’- ACAGGGCTCTGGAGACACAG -3’ |
| Human Tbx6 | 5’- AGCCTGTGTCTTTCCATCGT -3’ |
|  | 5’- AGGCTGTCACGGAGATGAAT -3’ |
| Human Oct3/4 | 5’- GACAGGGGGAGGGGAGGAGCT AGG -3’ |
|  | 5’- CTT CCC TCC AAC CAG TTG CCC CAA AC -3’ |
| Human Nanog | 5’- CAGCCCCGATTCTTCCACCAGTCCC -3’ |
|  | 5’- CGGAAGATTCCCAGTCGGGTTCACC -3’ |
| Human Sox2 | 5’- GGGAAATGGGAGGGGTGCAAA AGAGG -3’ |
|  | 5’- TTGCGTGAGTGTGGATGGGATTGGTG -3’ |
| Human T | 5’- ACCCAGTTCATAGCGGTGAC -3’ |
|  | 5’- CATTGGGAGTACCCAGGTTG -3’ |
| Mouse Rpl13a | 5’-GTGGTCCCTGCTGCTCTCAAG -3’ |
|  | 5’-CGATAGTGCATCTTGGCCTTTT -3’ |
| Mouse Pax3 | 5’-TCCATCCGACCTGGTGCCAT -3’ |
|  | 5’-TTCTCCACGTCAGGCGTTG -3’ |
| Mouse Tbx6 | 5’- CCCAACTATGCAGCCAACACT -3’ |
|  | 5’- CTGTGTGATCCTAGGGTTCTGGTA -3’ |
| Mouse Mesp2 | 5’- CTGAAAACCTTGGGAACAGGAT -3’ |
|  | 5’- GGCTCTTTCTAGGGACTGGTGTAA -3’ |
| Mouse Myf5 | 5’-CCACCTCCAACTGCTCTGA -3’ |
|  | 5’-GCTGTCAAAGCTGCTGTTCTT -3’ |
| Mouse Nanog | 5’-AAGTACCTCAGCCTCCAGCA -3’ |
|  | 5’ -CGTAAGGCTGCAGAAAGTCC -3’ |
| Mouse Oct3/4 | 5’-AGCTGCTGAAGCAGAAGAGG -3’ |
|  | 5’-TGGGAAAGGTGTCCCTGTAG -3’ |
| Mouse β-Actin | 5’-AGTGTGACGTTGACATCCGT -3’ |
|  | 5’- GCAGCTCAGTAACAGTCCGC -3’ |
| Mouse Nodal | 5’- ACGTTCACCGTCATTCCTTC -3’ |
|  | 5’- AGAGCATGAGCACATTGGTG -3’ |
| Mouse Mixl1 | 5’- CTACCCGAGTCCAGGATCCA -3’ |
|  | 5’- ACTCCCCGCCTTGAGGATAA -3’ |
| Mouse Gsc | 5’-GAGAAGGTGGAGGTCTGGTTTAAG -3’ |
|  | 5’-TTTGAGGACGTCTTGTTCCACTT -3’ |
| Mouse T | 5’-GCTCCCCTGCACATTACACA -3’ |
|  | 5’-TGACTGTAGCAGCCCCTTCA -3’ |
| Mouse Eomes | 5’- GGCCTACCAAAACACGGATATC -3’ |
|  | 5’- TTTCTGAAGCCGTGTACATGGA -3’ |
